# Experimental Analysis of Viral–Host Interactions

**DOI:** 10.3389/fphys.2019.00425

**Published:** 2019-04-11

**Authors:** Joseph Gillen, Aleksandra Nita-Lazar

**Affiliations:** Laboratory of Immune System Biology, National Institute of Allergy and Infectious Diseases, National Institutes of Health, Bethesda, MD, United States

**Keywords:** interactomics, host–pathogen interactions, proteomics, virus, immunity

## Abstract

Viral and pathogen protein complexity is often limited by their relatively small genomes, thus critical functions are often accomplished by complexes of host and pathogen proteins. This requirement makes the study of host–pathogen interactions critical for the understanding of pathogenicity and virology. This review article discusses proteomic methods that offer an opportunity to experimentally identify and analyze the binding partners of a target protein and presents the representative studies performed with these methods. These methods divide into two classes: *ex situ* and *in situ*. *Ex situ* assays depend on bindings that occur outside of the normal cellular environment and include yeast two hybrids, pull-downs, and nucleic acid-programmable protein arrays (NAPPA). *In situ* assays depend on bindings that occur inside of host cells and include affinity purification (AP) and proximity dependent labeling (PDL). Either *ex* or *in situ* methods can be reliably used for generating protein–protein interactions networks but it is important to understand and recognize the limitations of the chosen methods when developing an interactomic network. In summary, proteomic methods can be extremely useful for interactomics but it is important to recognize the nature of the method when designing and analyzing an experiment.

## Introduction

The interactions between viral and host proteins are responsible for all aspects of the viral life cycle; from infection of the host cell, to replication of the viral genome, and assembly of new viral particles. The analysis of these protein–protein interactions (PPI) is an emerging field in biology and can elucidate the critical pathways involved in immunity, cellular signaling, replication, and cellular division. In addition, viral–host interactions are a potentially potent target for antiviral treatments, such as the HIV-entry inhibitor maraviroc that inhibits viral entry by binding to the cellular receptor (CCR5) and preventing its binding to the viral glycoprotein (gp120) (Fatkenheuer et al., 2005).

Recent advances in molecular biology, mass spectrometry, and bioinformatics have increased the throughput of analysis while simultaneously decreased the false-positive rate of interactomic assay results, with an increased focus on separating the true-positives from the false-positives identified during mass spectrometry. The overall goal of this review is to analyze the most common methods of interactome identification with their benefits and drawbacks. In the end, the readers should be able to design an assay to acquire an interactomic dataset and to screen the dataset for the high confidence results along with the system implications of those results.

**FIGURE 1:**
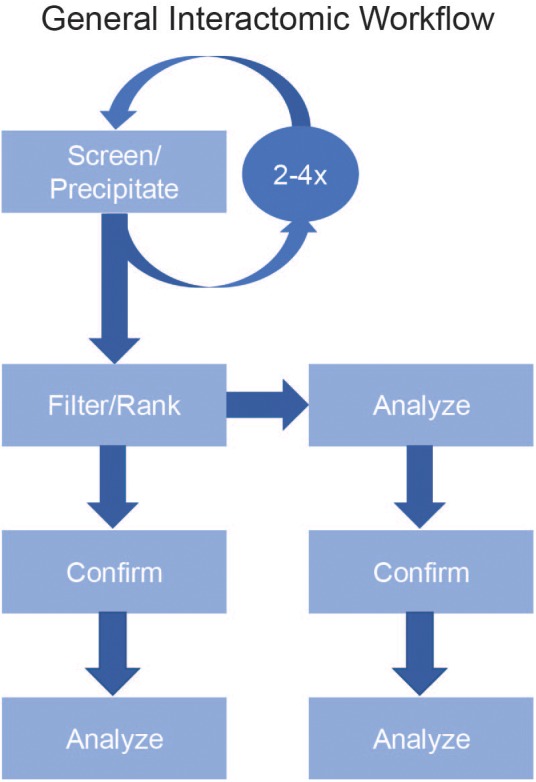
General Interactomic Workflow. To accurately map the interactome of a protein of interest (POI), begin by selecting your experimental method (Screen/Precipitate). To improve the recovery of low abundance proteins and to validate possible novel binding partners it is strongly suggested to repeat the assay two to four times then compile the results prior to filtering. To define the true-positives from false-positives, use a filtering process (Filter) followed by a ranking process (Rank) to generate a list of high confidence candidates. After ranking, it is equally acceptable to proceed directly to the data analysis step or to first confirm the previous results either by repeating the precipitation then using a different detection method (e.g., LC-MS/MS vs. western blot) or to use a new precipitation method (BioID vs. AP-MS).

The two main pipelines of acquiring interactomic datasets are *ex situ* binding assays [glutathione s-transferase (GST) pull-downs, yeast two-hybrids, and Nucleic Acid-Programmable Protein Array (NAPPA)] and *in situ* binding assays [affinity purification-mass spectrometry (AP-MS) and Proximity-Dependent Labeling (PDL)]. In general, these assays use a protein of interest (POI) as a bait for a pool of possible prey proteins. None of these assays are perfect for every protein nor are they exclusive. In fact, a common method of confirming high confidence results is to repeat the experiment using one of the other methods (de Chassey et al., 2008; Rozen et al., 2008; Liu et al., 2018; Figure 1).

While the addition of these techniques has increased the accumulation of interactomic data, one constant problem in interactomics is to identify the high confidence points in the data. To address this, labs have mapped the common false-positive proteins [contaminant repository for affinity purification-mass spectrometry data (CRAPome) (Mellacheruvu et al., 2013)] and generated screening programs to analyze the data and highlight the most consistently strong results [Significance Analysis of INTeractome (SAINT) (Choi et al., 2011) and Comparative Proteomic Analysis Software Suite (CompPASS) (Sowa et al., 2009)]. Once the high confidence results have been identified the question becomes how these interactions affect the cellular system. To address this, improvements in systems analysis have enabled researchers to quickly fit the identified proteins into pathways and identify biological processes strongly associated to the dataset (Search Tool for the Retrieval of Interacting Genes/Proteins (STRING) (Szklarczyk et al., 2015) and for viral-host PPI Viruses.STRING (Cook et al., 2018). Together, these advances in data analysis have decreased the chance of false-positive protein identifications and increased the depth of analysis for interactomic experiments.

## Methods for Acquiring the Database

To generate an interactome, the first decision is which assay to use. While there are many variations and modifications, most assays fall into two distinct classes: *ex situ* and *in situ*. *Ex situ* experiments are those where the interactions occur not in a host cell but instead occur either in a non-host cell or solution, while *in situ* experiments are those where the interactions occur inside a host cell. Generally, *ex situ* experiments can be adapted to screen large numbers of bait and prey combinations simultaneously, but the identified interactions can include artifacts due to the forced colocalizations or modified folding of the proteins being analyzed (Koegl and Uetz, 2008). In contrast, *in situ* experiments limit the effect of forced colocalization, but do not readily adapt to whole proteome screenings beyond a few bait proteins. Either class can be employed for most host or viral proteins but come with their own benefits and drawbacks.

### *Ex situ* Binding Assays

All *ex situ* assays rely on generating your POI outside of the viral-infected host cells then measuring its binding to host proteins. Because the POI is expressed in the absence of the viral-infected host, these assays can limit exposure to dangerous pathogens and decrease the biosafety level (BSL) required for analysis. In contrast, interactions identified in these procedures may be completely artificial and unrelated to the protein’s functional role (Koegl and Uetz, 2008), as such experimental designs using *ex situ* approaches should consider the addition of an *in situ* confirmation step or an additional *ex situ* experiment designed to test this result.

#### Yeast Two-Hybrid

**FIGURE 2:**
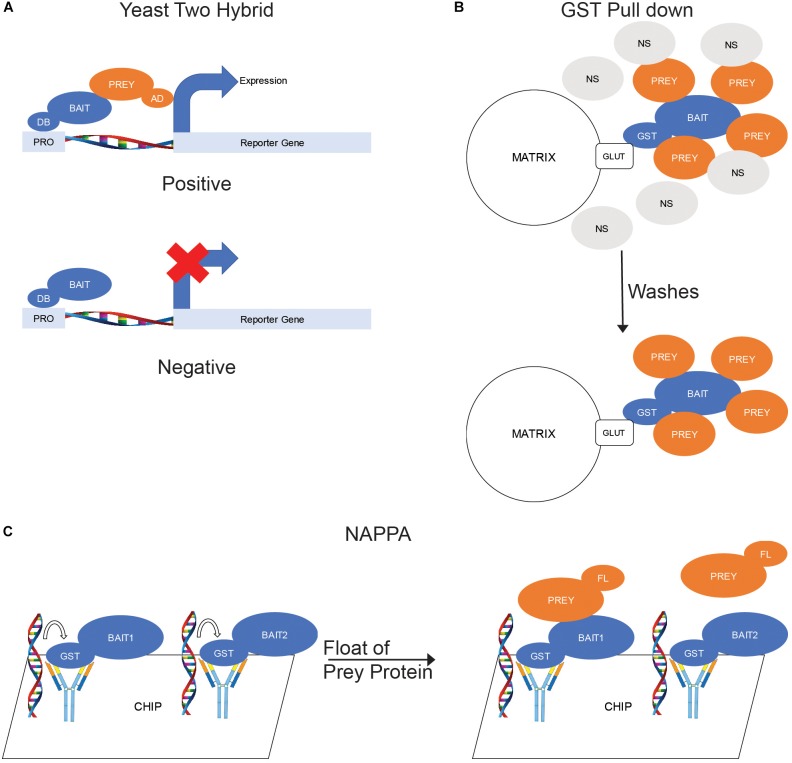
*Ex situ* Binding Assays. **(A)** Yeast two hybrid. The bait proteins are fused with a DNA-binding domain (DB) and coexpressed in yeast cells with prey proteins fused with a transcription activating domain (AD). When the bait binds a prey, the DB and AD domains become positioned to activate transcription of a reporter gene. **(B)** GST Pull down. The bait protein is fused with glutathione s-transferase (GST) and purified by exposure to a matrix labeled with glutathione (GLUT). The matrix is then mixed with cellular lysates to allow for prey proteins to bind to the bait. **(C)** Nucleic acid-programmable protein arrays (NAPPA). DNA sequences encoding a library of proteins are fused to a chip then *in vitro* expression used to produce proteins. The proteins are captured by antibodies also fused to the chip to produce a protein array. The prey protein, which is fluorescently labeled prior, is floated over the chip to allow binding to the baits.

The most common *ex situ* binding assay is the yeast two-hybrid assay. In a yeast-two hybrid experiment, one protein is constructed as a chimera fused to the DNA-binding portion of a transcription factor (bait) while a second protein is constructed as a chimera fused to the activation domain of a transcription factor (prey). Both proteins are then expressed in yeast cells and, if binding occurs, an active transcription factor forms and a reporter gene is expressed (Figure 2A).

The benefits of this assay include the use of protein libraries to map the binding of a single POI to multiple proteins in parallel. Once a PPI has been identified, the assay can be used to map the binding sites of two proteins by constructing baits and preys from the fragments of the proteins. In addition, the yeast two-hybrid can be used for drug discovery by treating cells containing appropriate bait-prey combination and recording the effect of the treatment on reporter gene expression.

The drawbacks of this assay include the cloning of the POI into an appropriate bait or prey construct, which takes time. In addition, the fusion of the transcription factor domains may affect the overall protein fold, which can affect the presentation and/or function of binding sites. Also, the bait-prey binding must occur in the nucleus for reporter gene expression and this forced colocalization can allow artificial binding events to occur. While yeast two-hybrid libraries can be expansive, bindings are limited to only those proteins in the library and are usually limited to only binary interactions. This prevents the addition of critical cofactors such as accessory proteins and modifying proteins. Lastly, yeast two-hybrid assays have a high percentage of false positive results (Koegl and Uetz, 2008; Cook and Jensen, 2018).

An additional drawback of the standard yeast-two hybrid is that while it can screen the interactions of a soluble POI with a library of bait proteins, membrane-bound or transmembrane proteins often cannot be analyzed. A novel method for analyzing membrane protein interactomes is the split ubiquitin membrane yeast two hybrid, also known as MYTH or MbYTH. In a MYTH screen, one protein is bound to the *N*-terminal portion of a ubiquitin moiety (Nub) and the second protein is bound to the C-terminal portion (Cub) linked to a reporter protein. When the two proteins bind, the Nub and Cub regions join and form a full ubiquitin moiety that binds to ubiquitin-specific proteases that cleaves the reporter protein from the complex producing a positive result (Stagljar et al., 1998).

Despite these drawbacks, yeast two-hybrid assays have been used to map the host-viral interactomes of Epstein-Barr virus (EBV) (Calderwood et al., 2007), Hepatitis C virus (HCV) (de Chassey et al., 2008), and Kaposi’s sarcoma-associated herpesvirus (KSHV) (Uetz et al., 2006). In EBV, the study identified 40 EBV proteins binding to 112 human proteins with 173 interactions along with a network of 60 viral-viral PPIs. For HCV, the researchers identified 314 interactions between the 11 HCV proteins and the host protein library. In KSHV, the authors used 89 KSHV proteins and mapped 123 viral–host interactions. In all three cases, the screens identified bindings between viral proteins and host immunity proteins, which may suggest the viral protein is an inhibitor of the immune response, and between viral proteins and host signaling proteins, which may be important for viral replication. In addition, MYTH was used to analyze the interactome of the Tombusvirus protein p33 and identified 19 host protein binding partners, including the E2 ubiquitin-conjugating enzyme Cdc34p, which was found to be required for viral replication (Li et al., 2008).

#### GST Pull-Down

In addition to yeast two-hybrid methods, pull-downs are a very common form of *ex situ* binding assay. Pull-downs involve the use of purified proteins as the bait and can use either purified proteins or cellular lysate as the prey. One of the most common types of pull-down experiment are GST pull-downs. In a GST pull-down, the bait protein is expressed as a chimera fused to a GST domain in a suitable culture, usually *Escherichia coli* but can also be *Saccharomyces cerevisiae* or insect cells. The GST-fusion protein is then purified by the binding of the GST to a glutathione-labeled matrix, often agarose beads. The purified proteins can then be eluted from the matrix by exposure to free glutathione then mixed with the prey proteins prior to binding to a fresh matrix or the matrix-bound bait can be mixed with the prey proteins. The matrix is washed to remove any non-specific interactors and the prey proteins eluted for analysis (Figure 2B).

The benefits of this assay include the ease of use and low cost. Also, the concentration of the purified protein can be adjusted to improve sensitivity and varying concentrations can be used to estimate the binding affinity of proteins (Lapetina and Gil-Henn, 2017). Another benefit is that the purified proteins can be generated in bacterial, yeast, or insect cells and thus may not be cleaved or degraded prior to analysis.

The drawbacks of this assay include that to produce the protein to be purified requires cloning the protein, which takes time, and the fusion of a GST-domain onto your POI, which can affect the overall fold and binding abilities of the protein. Like yeast two-hybrid, all the binding reactions occur outside of the proteins’ native environments and thus may not occur in the cells naturally. Additionally, the extraction of the proteins from their native environments could lead to misfolding, which could affect its binding to other proteins. Another common issue with the expression of GST-tagged proteins can lead to the formation of inclusion bodies that prevent the purification of active proteins. These inclusion bodies can often be solubilized by denaturation followed by *in vitro* folding, but the artificially folded proteins may not have their native conformation and often lack any posttranslational modifications. In addition to folding issues, the concentrations of both the bait and prey proteins may not be consistent with those found in cells, which can enhance the recovery of artificial bindings.

Glutathione s-transferase pull-downs have been very useful in the development of interactomes. For the HIV-1 Tat protein, researchers used GST pull-downs to isolate the Tat-interacting protein complexes from the nuclei of T-cells and found that Tat can stably bind proteins involved in regulating transcription, regulating translation, and modulating the structure of chromatin (Gautier et al., 2009). In Hepatitis B virus, the HBx protein interactome was mapped by using mass spectrometry analysis on the proteins eluted from GST-HBx-coated beads. This identified apolipoprotein A-I (apoA-I), which is known to function in the lipid and cholesterol metabolic pathways, as a strong binding protein. Furthermore, the researchers showed that increasing the amount of apoA-I by transfection had a protective effect on HBV-infected mice, suggesting that interfering with HBV’s manipulation of apoA-I may be a possible target for anti-HBV treatments (Zhang et al., 2013).

#### NAPPA

A more recent version of *ex situ* binding assay is the Nucleic Acid-Programmable Protein Array (NAPPA). A chip-based assay, NAPPA uses *in vitro* expression of proteins encoded by nucleic acids fused to the chip to generate a protein chip studded with a library of proteins. These bait proteins, which are linked to the chips by covalently bound antibodies, are then exposed to purified prey protein labeled with a fluorescent marker. After washing, a spot of fluorescence will be detectable where the prey protein has bound to the immobilized bait (Tang et al., 2017; Figure 2C).

The benefits of this assay include that adaptability of the microarrays, which can be printed with fragments or mutants of proteins to map the sites and residues required for binding to the POI. In addition, the *in vitro*-expressed proteins on the chip can include proteins with high degradation rates or proteins that cannot be expressed in other systems.

The drawbacks of this assay include that generating the chip can be expensive, time consuming, and every binding requires its own chip. In addition, the *in vitro* expression means that the protein will not be modified like it would be in a cellular environment, which could affect its fold and affinity to binding partners.

Though a relatively new procedure, NAPPA has been used to map the interactomes of all five rubella viral proteins. In addition to the three previously reported proteins, the researchers identified 55 candidate host proteins and were able to predict networks for each of the viral proteins that may provide information about infection and replication (Yu et al., 2014). In another study, the researchers examined the interactomes of the *Legionella pneumophila* effector proteins (SidM and LidA). A Gram-negative pathogenic bacterium, *L. pneumophila* injects nearly 300 effector proteins into host cells and infections can cause legionnaire’s disease. Interestingly, both proteins showed strong affinity to multiple Rab GTPases, a class of proteins which are known to affect membrane trafficking (Yu et al., 2015).

### *In situ* Binding Assays

In contrast to *ex situ* binding assays that occur with proteins outside of their host cells, *in situ* assays all involve the binding of the POI to host proteins inside of the cell. The two classes of *in situ* binding assays are AP-MS and PDL. Because the binding occurs inside of host cells, the number of artificial interactions is limited and the modifications on the proteins can be closer to the state found during infection. These factors can be beneficial during an interactomic analysis, but each version of these binding assays also has its own weaknesses.

#### Affinity Purification-Mass Spectrometry

One of the most common and adaptable methods for studying protein interactomes, AP-MS can be done with dozens of modifications and variations. In general, AP-MS procedures begin by solubilizing and subsequentially precipitating the POI, along with any proteins that are stably associated to the POI, by means of an antibody or binding motif linked to a support matrix. The matrix is washed to remove any non-specific binding proteins and the bound proteins are digested then analyzed by liquid chromatography-mass spectrometry/mass spectrometry (LC-MS/MS). A common benefit of AP-MS experiments is their relative ease of use. Often the most difficult portion of the assay is deciding which type of affinity to use for precipitation. In addition, depending on the type of analysis used following the precipitation, the sizes of complexes can be analyzed following the initial precipitation. A shared drawback is that AP-MS experiments often precipitate not only the proteins bound to the POI, but also those bound to the tubes, matrix, pipets, etc. To address this, a control sample is used where the assay is repeated without adding the antibody required to precipitate the POI and compared to the samples containing the antibody. Additionally, the lysis of the samples prior to AP-MS requires the addition of detergents to open the cell membranes and solubilize the protein complexes. Depending on the binding strengths of the proteins, even mild detergents may dissociate binding partners from the POI. In this review we will focus on three of the most common versions of AP-MS: native, GFP-tagged, and tandem.

##### Native AP-MS

**FIGURE 3:**
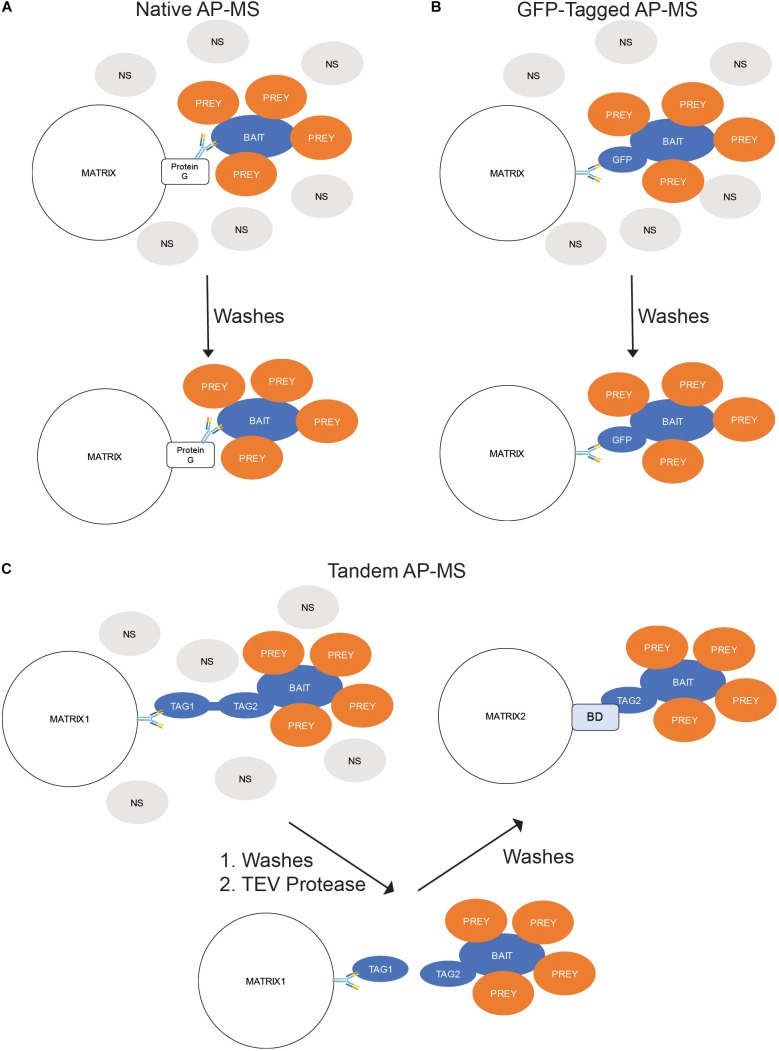
*In situ* Binding Assays – Affinity Purification-Mass Spectrometry (AP-MS). **(A)** Native AP-MS. Cellular lysates containing the protein of interest (POI) are exposed to an antibody toward the POI. The resulting immunocomplexes are precipitated from the lysate by exposure to a Protein G-labeled matrix while the non-binding (NS) proteins are washed away. **(B)** GFP-Tagged AP-MS. The POI is expressed in cells as a fusion with green fluorescent protein (GFP). The cells are lysed; and the GFP-containing complexes precipitated by an anti-GFP antibody-labeled matrix while the NS proteins are washed away. **(C)** Tandem AP-MS (TAP-MS). The POI is expressed in cells as a fusion with two tags separated by a tobacco etch virus (TEV) protease site. The cells are lysed; and the lysate mixed with antibodies toward the distal tag (Tag1). The resulting immunocomplexes are precipitated from the lysate by exposure to a Protein G-labeled matrix while the non-binding (NS) proteins are washed away. The tag is then cleaved by adding TEV protease and the complexes precipitated by the proximal tag (Tag2) binding to a binding domain (BD)-labeled matrix.

Native AP-MS is dependent upon binding the POI with an antibody. The antibody can be precipitated by exposure to a Protein A-, Protein G-, or Protein A/G-bound to a matrix. Proteins A and G are antibody-binding proteins isolated from bacteria while Protein A/G is a chimeric version of both proteins. All three can be readily purchased covalently linked to agarose or magnetic beads. Prior to purchase, the researchers should confirm which type of bead is best for their antibody; some antibodies bind best to one over the others. After the binding of the antibody-bound proteins to the beads, the non-specific and unbound proteins are removed by washing, then the precipitated proteins are eluted and analyzed by MS (Figure 3A).

The benefits of this assay include the POI can be precipitated from viral-infected host cells including patient samples. The co-precipitated proteins will include proteins that bind either directly or indirectly to the POI and when combined with LC-MS/MS analysis nearly any host protein can be identified. In addition, the MS analysis can also indicate the presence of many post-translation modifications, including phosphorylation.

The drawbacks of this assay include the requirement of strong binding for a protein to co-precipitate with the POI – weak binding or interacting proteins will be lost – and the potentially extensive optimization required to precipitate the POI along with its binding partners. Native AP-MS requires an antibody toward the POI. These may be available from vendors but often require inhouse generation, which takes time and increases cost (Cristea et al., 2005). Another concern with POI-specific antibodies is that while the antibody may give a strong and clean result during assays like western blot or ELISA, it is possible that the antibody will not function well during an AP-MS experiment. This could be due to the epitope being obstructed by the fold of the protein in cells, the binding of proteins to the region that contains the epitope, or the addition of PTMs to the residues in/around the epitope (Gillen et al., 2015).

While native AP-MS can be difficult and requires screening of antibodies for their efficiency, it has been used to map the interactomes of several viral proteins. Using a mouse monoclonal antibody, researchers identified over 50 host and viral proteins that bind to the herpes simplex 1 virus (HSV-1) protein ICP8, a DNA-binding protein required for viral genome replication (Taylor and Knipe, 2004). In Kaposi’s sarcoma-associated herpesvirus, researchers used a panel of in-house generated monoclonal antibodies to identify the binding partners of ORF45, a required viral protein that inhibits the innate immune response and assembles into the viral particle. In addition, by mapping the epitopes of each antibody and using a panel to precipitate ORF45-containing complexes, the researchers were able to map the binding sites of the ORF45-binding proteins during the AP-MS experiment (Gillen et al., 2015).

##### Green fluorescent protein (GFP) tagged AP-MS

Because antibodies toward a POI are often unavailable and would require time and money to generate, a common solution is to generate a protein chimera fused with a tag that contains and well characterized epitope. These tags can be range from short epitopes like FLAG or hemagglutinin (HA) to large molecules like GST. One of the most useful tags is green fluorescent protein (GFP). As a well-studied protein, GFP has several commercially available antibodies that can precipitate it during AP-MS experiments. The overall precipitation procedure is identical to that of native AP-MS; the cells are lysed, an antibody added to the mix to create antibody-bound protein complexes, and those complexes being precipitated by the affinity of the antibodies to Protein A-, G-, or A/G-labeled beads. The main difference between native and GFP-tagged AP-MS is the requirement of expressing a GFP-tagged version of the POI in your target cell. This can be accomplished by transient transfection, stable expression (i.e., retroviral induction), or genome editing (i.e., CRISPR) (Figure 3B).

The benefits of this assay include the large number of commercially available anti-GFP antibodies, including some that come covalently linked to beads so the use of antibody binding beads can be avoided. The common use of this tag also means that the amount of optimization can be reduced since the buffer conditions to compatible with the binding of GFP to its antibodies is readily available. An added bonus of a GFP-fusion is that the GFP signal can be used to confirm expression of the POI prior to lysis and to map the localization of the POI, which can be used to filter out potential false positives. In addition, the GFP molecule can be expressed unfused to the POI to act as a control sample to identify proteins that bind to the beads, tubes, GFP, or antibodies and not the POI.

The drawbacks of this assay include that cloning the POI into a GFP-tagged vector could lead to changes in the fold and function of the POI, which can affect its binding partners. Also, the GFP tag can affect the localization of the POI in cells, which can lead to its binding to new partners. Additionally, the most common way to express the GFP-tagged POI is transient transfection, which can lead to unnatural expression levels of the POI and limits the availability of host cells because not all host cells are compatible to transfection. Lastly, while the GFP-tag is easy to add to a POI in a plasmid using modern cloning techniques, this tag will not be present on the POI in natural samples such as those gathered from patients. This means that GFP tagged AP-MS cannot be used on wild type virus.

With the recent improvements in molecular cloning, GFP tagged AP-MS has become one of the most common AP-MS techniques. GFP AP-MS was used to identify the binding partners of the human respiratory virus (HRSV) RNA polymerase complex and found that replication of the viral genome requires the binding of heat shock protein 90 (HSP90) to the polymerase by stabilizing the viral L-protein (Munday et al., 2015). In HSV-1, a recent study found that the interferon-inducible protein X (IFIX) is bound by several E3-ubiquitin ligases during viral infection and this leads to the degradation of the host immunity protein during HSV-1 infection. This is critical for viral replication because IFIX suppresses the expression of viral immediate-early and early genes following infection (Crow and Cristea, 2017).

##### Tandem AP-MS

In most AP-MS experiments a single affinity mechanism is used to link the POI-containing complexes to the matrix. An alternate procedure is two use two affinity mechanisms sequentially to precipitate the POI-containing complexes. Termed tandem AP-MS (TAP-MS), this process begins by cloning a tag containing two binding regions separated by an endoproteinase site (usually the tobacco-etch virus (TEV) motif). This tag allows for a stage one precipitation using the distal tag followed by cleavage of the POI-containing complexes from the first matrix by adding TEV protease. The released proteins are then precipitated using the proximal tag and the POI-containing complexes eluted from the second matrix (Figure 3C).

The benefits of this assay include the increased selection of using a two-step precipitation which can limit the amount of false positive results by limiting the accumulation of matrix binding proteins. This benefit can be enhanced by using tags that involve differing mechanisms, such as the binding of antibody to antibody-binding beads followed by the binding of streptavidin to biotin. Similar to a standard tagged AP-MS, TAP-MS requires less optimization since the bindings of the tags to the matrix is well established prior to use. Additionally, the extension of the tag from the POI reduces the chances of the tag being blocked during the precipitation by a binding protein or fold.

The drawbacks of this assay include the need for twice the material for two-step purification process than for one-step process. The purification process could also require advanced equipment such as a high-performance liquid chromatograph (HPLC) or specialized spin columns. The addition of the tag to the POI could also affect the fold of the protein leading to artificial localization and binding partners. In addition, because the tag must be cloned onto the POI and the resulting chimera expressed in host cells, the procedure cannot be done on patient samples or wild type virus and the expression levels of the POI can be vastly different from that found in nature.

Epstein-Barr Virus-encoded nuclear antigen 5 (EBNA5) is a protein that acts with the EBV protein EBNA2 to activate the expression of the EBV oncogenic protein latent membrane protein 1 (LMP1) and can affect the processing of mRNA. Using TAP-MS, researchers found that EBNA5 binds to several proteins involved in pre-mRNA processing, protein folding, protein degradation, and transcription (Forsman et al., 2008). In a broad screen of viral proteins known to suppress the innate immune response, researchers used TAP to compare the interactomes of 70 proteins from 11 different viral families including both RNA- and DNA-encoded genomes. After comparing the interactomes, the researchers identified 579 cellular proteins and mapped the most common binding partners of viral proteins are proteins that act in multiple pathways (Pichlmair et al., 2012).

#### Proximity-Dependent Labeling

A recent addition to the interactomics toolkit is PDL, a process where an enzyme chemically labels a protein(s) in close proximity to the POI. For a PDL assay to be a viable experiment the labeling must not be restricted by any other factor than distance from the POI. The labeling must also be able to survive the precipitation process until the non-specific proteins have been separated from the coprecipitated fraction. In addition, it would be beneficial if the mechanism of the PDL had little or no background labeling to enhance the difference between the non-specifically labeled proteins and the interacting proteins. The first system to fulfill these requirements is the biotinylation based BioID system.

##### BioID

**FIGURE 4:**
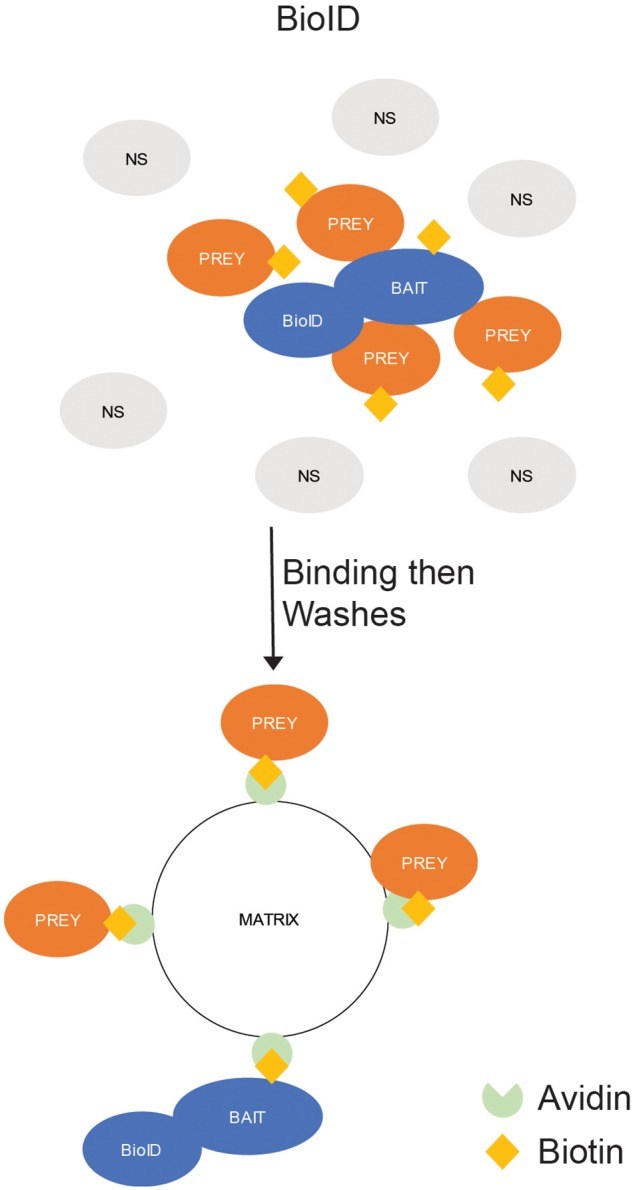
*In situ* Binding Assays – Proximity-Dependent Labeling (PDL). BioID. The protein of interest (POI) is expressed in cells as a fusion with a promiscuous biotin ligase (BioID). The ligase labels all proteins in close (10–15 nm) proximity to the POI with biotin. The labeled proteins are precipitated by exposure to an avidin-labeled matrix, which strongly binds biotin-labeled proteins, and the unlabeled (NS) proteins are washed away.

Biotinylation is the process of covalently binding a biotin molecule to a lysine by a biotin ligase. While biotin can be linked to lysines by a ligase, it can also be bound by avidin, streptavidin, and NeutrAvidin. This allows a biotinylated protein to be precipitated from a solution by mixing the protein solution with avidin-bound beads or a similar matrix. In addition, the binding of biotin to avidin is one of the strongest non-covalent bonds ever studied, which allows for the use of extremely stringent wash strategies. To use biotinylation in interactomics, mutants of naturally occurring biotin ligases have been generated to produce a promiscuous biotin ligase (termed BioID) that would biotinylate proteins in close proximity of the BioID-fused protein. The biotinylated proteins are then precipitated by binding to an avidin matrix (Roux et al., 2018; Figure 4).

There are many benefits of this assay. The biotin ligase will label any proteins in close proximity to the POI inside the cell including both strong binding proteins and weak interacting proteins. In addition, the binding affinity of biotin to avidin is one of the strongest in nature and thus allows for extremely stringent washing of the matrix to remove non-specific binding proteins. While the original BioID had a slow reaction rate and required hours of labeling time to generate enough biotinylated proteins for a proper analysis, newly developed versions of the ligase termed TurboID and MiniID offer enhanced biotinylation (Branon et al., 2018).

It is worth mentioning that several other proximity labeling methods have been successfully developed and used in other types of studies, so they may prove useful for identifying virus–host interactions in the future. These include the ascorbate peroxidase assays (APEX and APEX2) (Martell et al., 2012; Lam et al., 2015); and the horseradish peroxidase (HRP)-based assays: biotinylation by antibody recognition (BAR) (Bar et al., 2018); enzyme-mediated activation of radical sources (EMARS) (Jiang et al., 2012); and selective proteomics proximity labeling assay using tyramide (SPPLAT) (Rees et al., 2017). Examples of their use in the numerous areas of biology have been a subject of some excellent reviews (including Rees et al., 2015; Han et al., 2018).

The drawbacks of this assay include the possible effects of the cloning of the chimeric fusion with a biotin ligase on the activity and localization of the POI. When designing the chimera, it is also important to consider the positioning of the biotin ligase. This is due to the nature of the labeling – the biotin ligase has a range of 10–15 nm and cannot label across plasma membranes. If the prey protein binds the bait distant from the fused biotin ligase, then the assay can produce a false-negative result. In the case of transmembrane proteins, fusing the ligase to the portion of the protein on one side of the membrane can provide a completely different interactome from the other. In addition, the 10–15 nm labeling range means that not only those proteins that bind to or interact with the POI may be labeled but also those proteins in close proximity to the POI at the time of labeling, leading to false positive results.

Using BioID, researchers were able to generate interactomes for all ten polypeptides encoded by the Zika virus (ZIKV). In total, 1224 human proteins were identified as interacting with Zika viral proteins. An analysis of the proteins identified many pathways previously reported as required for viral infection, including translation, protein processing, vesicle trafficking, and lipid metabolism, along with several novel interactors (Coyaud et al., 2018). In EBV, researchers used BioID to identify the binding proteins of latent membrane protein 1 (LMP1), a viral oncogenic protein. In total, over 800 proteins were identified as binding to LMP1. While many of the binding proteins had been previously identified, several novel proteins were identified in the BioID assay and not in an AP-MS experiment done in parallel (Rider et al., 2018).

## Data Analysis

While either *ex situ* or *in situ* binding assays can generate lists of possible binding proteins, it is important to be able to filter the false-positives from the true-positives. While yeast two-hybrid and NAPPA screens are relatively straight forward to analyze, with a simple comparison to a control bait as a useful comparison to identify preys that specifically interact with your POI (Koegl and Uetz, 2008), screens that rely on MS analysis have increased noise that can obscure your signal. A common approach to separate the signal from the noise is to rank the positives based upon a confidence score so the researcher can pursue the strongest candidates, which if properly filtered will consist of the majority of true-positive results. Once the researchers have sorted the data into a list of strong candidates, an important question is how these candidates may interact and what pathways, systems, or known interactions link these candidates. These linkages could suggest the roles of the viral-host protein-protein interaction, illuminate the role of these proteins in the viral life cycle, and provide new targets for antiviral treatments (Nesvizhskii, 2012).

### Filtering of False-Positives

One of the most difficult aspects of an interactomic experiment is to filter the non-specific binding proteins from the specific binding proteins. These false-positives can be due to binding of proteins to the antibodies, matrix, tubes, or even tips used during the experiment. In addition, highly abundant proteins can survive the washes used to remove unbound proteins due to their large starting number, which can mean that 5 or more 10-fold washes may not be enough to completely remove the protein from the remaining buffer around the beads. These false-positive proteins can mask the presence of the true-positive proteins, those that bind to the POI.

#### Pre-processing

The simplest method for removing false-positive proteins is to compare the experiment results to a control assay done in parallel using all of the same materials and methods. By combining this method with a list of known commonly recovered proteins, it is possible to limit the list of binding candidates to a more manageable size. However, while the comparing the results of an interactomic experiment to a control is a useful first step, it is an extremely rough method for identifying possible candidates and can be biased due to the large amount of user selection used. To begin with, this comparison does not account for reproducibility of a candidates. Also, by simply discarding those proteins that appear in a control sample, the researchers will lose proteins that may coprecipitate at a low level in control samples but are strongly coprecipitated from the POI-containing samples (Morris et al., 2014).

#### CRAPome

In a small scale interactome, the data may require only a filtering out of known false-positive proteins to generate a list of the POI-specific proteins. To identify the common proteins that precipitate during AP-MS experiments independent of the POI target, researchers from over twenty institutes compiled a list of the proteins precipitated from their control samples. This list was sorted to match the results with the characteristics of AP-MS used (type of beads, host cells, etc.) and to compare the quality of the identification by the LC-MS/MS (number of peptide spectral matches (PSMs), intensity of signal, etc.). After this sorting, the high confidence results were compiled into a searchable database of known non-specific interactors called the contaminant repository for affinity purification (CRAPome) (Mellacheruvu et al., 2013).

A free to use tool, CRAPome acts as an initial filter of AP-MS and BioID data that can remove the common non-specific interactors found in *H. sapiens, S. cerevisiae*, and *E. coli* results based upon the previous results of labs from across the globe. In addition, the software can use a user provided control sample to generate a fold change score (FC) of the biological replicates for each protein in the data set. The program then compares the average FC (FC-A) to the geometric mean FC (FC-B) to generate a graph of with the FC-A score in the Y-axis and the FC-B score on the *X*-axis. The end result of this analysis is that the weakest candidates are closest to the origin and the strongest are the furthest away.

### Ranking True-Positives

Once you have removed the common false-positives, it is important to sort the list based upon a set of criteria designed to target the potential high-confidence true-positives. To rank the possible candidates, the results from the LC-MS/MS runs can provide invaluable information beyond the identification of the proteins in the sample. To make better use of this additional data generated by the LC-MS/MS, researchers have generated several software packages that compare the results from controls to samples and then generate a score for each candidate protein. Three of the most commonly used programs are the CompPASS, SAINT, and MiST.

#### CompPASS

To move beyond a simple presence v absence binary evaluation the first piece of data to consider is the number of PSMs or total spectral counts (TSC) for each protein. By generating a table of the identified proteins with the number of PSMs for each, the candidate proteins can be ranked by comparing the TSC of each protein. To accomplish this, researchers developed the CompPASS, a free to use tool that uses a standard (Z) score and a newly developed Normalized Weight D (NWD) score. The Z score is a traditional statistical measurement of deviation in the number of spectral counts per protein in the experimental samples over the whole dataset. The NWD score is an improvement on the Z score because it includes reproducibility and accounts for differences between amount of each unique protein (Sowa et al., 2009). A benefit of CompPASS is that the program can analyze multiple bait protein results simultaneously. Unfortunately, if you use multiple bait proteins you must be sure that the two proteins do not have overlapping interactomes as the Z and NWD scores cannot account for shared true-positives (Morris et al., 2014).

#### SAINT

While CRAPome uses a comparison between a control sample and an experimental set and CompPASS uses a comparison of all the proteins found in the experimental set, a combination of both could be a potentially more effective process. The combination of both factors is the basis of the significance analysis of interactome (SAINT). During a SAINT analysis, the TSC of each protein in the experimental and control datasets, along with the TSC of the bait protein found in each dataset, are analyzed using Bayesian statistics to generate a plot of the spectral counts versus the estimated distribution. The relationship between two points is then analyzed to probability of interaction score ranging from 0 to 1. Because the SAINT score is based upon a comparison of the experimental and control datasets, a SAINT score can also be calculated using peptide ion intensity along with other continuous variables (Choi et al., 2011). Benefits of SAINT are that probability score is kept between 0 and 1, which are easier to understand and evaluate between runs, and the use of a control sample, which accounts for lab-specific differences in the results. Drawbacks of SAINT are that it does not handle secondary-binding proteins well, especially when the secondary protein has varying results between replicants, and that requirement of controls (Nesvizhskii, 2012).

#### MiST

Mass spectrometry interaction statistics (MiST) is a scoring system for interactome results that was designed to target viral–host interactions. The basis of the MiST score is to compare the abundance of the protein in the elution, the reproducibility of the result from run to run, and the specificity of the recovery of the prey based on the bait. While SAINT uses a two-axis comparison to plot the data as the basis of its analysis, MiST uses a three-axis comparison to produce a single MiST score that ranges from 0 to 1 (Jager et al., 2011). As a benefit, the MiST score is an easy to analyze score that accounts for more aspects than most other scores. A drawback of the MiST score is the complex nature of the process. As of this writing MiST exists as an R package and requires some knowledge of R to use fully. Two step-by-step instructions on using MiST were published by Morris et al. (2014) and Verschueren et al. (2015).

### Analysis of Interactome Results

Once an interactome is established the next question is what processes or pathways are affected by the binding of the proteins. With the large amounts of PPI identified in the literature, many proteins have known binding partners in at least one or more species. By comparing the genomes or proteins in the database between species it is also possible to generate a network of possible connections for even unstudied proteins. One of the most expansive and potently useful is the Search Tool for the Retrieval of Interacting Genes/Proteins (STRING). Another useful tool that builds on the STRING suite, while enhancing the coverage of interactions to include not only host PPI but also host-viral and viral-viral PPI, is Viruses.STRING. While both of these programs are very useful tools for analyzing PPI, it is important to remember that databases are only as good as the data entered into them. If the data entered is incomplete or incorrect then the interactions listed will also be flawed. This requires the researchers to always consider the confirmation of novel interactions or loss of a previously reported interaction.

#### String

The STRING database was developed as a means to give a critical assessment of PPI and to compile reported direct and indirect interactions. Based upon a series of eight scoring algorithms, STRING attempts to consider all possible ways of predicting interactions between proteins. Some of the eight scores are the homology score [uses the ortholog assignments in the Clusters of Orthologous Groups (COGs)], the co-expression score [uses microarray data along with known interaction pathways (KEGG)], and the experiments score (uses previously reported interactions imported from up to six PPI databases) (reviewed in Armean et al., 2013). In addition to its numerous scoring features, STRING has over 2000 organisms in its database, which means that nearly any interactomic dataset can be analyzed using the STRING database. Once a set of proteins is uploaded to STRING, the program will compute the PPI enrichment *p*-value, which indicates the confidence that STRING can link your protein and its identified binding partners to an indicated pathway. When used on a pathway known to be involved with your POI, the *p*-value can indicate the quality of your precipitation. In addition, STRING reports an enrichment analysis of the dataset based on gene orthologies, Kyoto Encyclopedia of Genes and Genomes (KEGG) pathways, and domains (Szklarczyk et al., 2015). The combination of this analysis with the precipitation results can be used to generate the pathways and processes dependent upon the viral protein, a strong benefit for an interactomic analysis. STRING does have some drawbacks though including that the process is gene-dependent and therefore ignores protein isoforms, alternative splicing, and post-translational modifications (Cook et al., 2018).

#### Viruses.STRING

While STRING has been extremely useful in analyzing PPI in a host species, the database does not account for the interspecies interactions found in viral-infected hosts. While innumerable studies have been conducted to map the interactomes of viral proteins, there have been very few suitable databases that compile this data and allow for its analysis. Recently, a new modified version of STRING named Viruses.STRING attempted to address this deficiency. Building on the methodology used to develop the STRING database, Viruses.STRING was built by text-mining the available scientific literature along with incorporation of experimental data from several common public databases prior to scoring based on the KEGG database. Once the pathways were established in one host-viral set, the interactions were then compared to orthologous in related species of both hosts and viruses. The end result is a database containing over 170 thousand PPI from 239 viruses that infect 319 host species. Similar to STRING, Virus.STRING allows the researches to map their proteins into GO-term pathways and provides a FDR *p*-value for each pathway. While this database could be a significant tool for analyzing host-viral PPI, the database is dependent upon published PPI results to develop its networks. In addition, while host proteins (and thus host-host PPI) change, mutate, or evolve at a slow rate, viral proteins can rapidly mutate and as such orthologous proteins from related viruses may not retain the same interactomes even when comparing between strains of a single viral species (Cook et al., 2018).

## Summary

Viral-host PPI are responsible for every aspect of infection and thus the root cause of all viral diseases. To analyze the binding of proteins researchers have developed several methods of interactomic analysis. These methods can be divided into two distinct classes: *ex situ*, or those methods where the binding occurs outside of their natural localization, and *in situ*, or those methods where the binding occurs in their natural localization. Once the results of the assay are compiled, the filtering of the true-positives from the false-positives followed by the ranking of the true-positive results to generate a high confidence protein target list is critical to efficiently study the role of the viral protein in the cell. These high confidence protein targets can then be further analyzed with pathway analysis tools, to generate a systems map of the viral life cycle with important nodes clearly defined. By targeting the critical nodes for the virus, we can develop new avenues of antiviral therapy and patient care.

## Author Contributions

JG and AN-L conceived the study and reviewed and edited the manuscript. JG drafted the manuscript. AN-L supervised the study.

## Conflict of Interest Statement

The authors declare that the research was conducted in the absence of any commercial or financial relationships that could be construed as a potential conflict of interest.
